# St. Bartholomew's Hospital Committee of Inquiry

**Published:** 1903-03-07

**Authors:** 


### ST. BARTHOLOMEW'S HOSPITAL COMMITTEE OF INQUIRY.

This committee, which was appointed on January 19th, has been sitting for some time at the hospital in Smithfield, Their first meeting was on February 16th, when evidence was taken upon the question of the retention of the hospital on its present site or its removal therefrom. After a long sitting the committee adjourned until Friday, February 20th, when Sir W. Hart Dyke, M.P., presided. Among the witnesses examined were Captain Nott-Bower, Commissioner of Police for the City of London; Sir Edmund Hay Currie, secretary of the Hospital Sunday Fund; and Mr. W. H. Cross, clerk to the Governors of St. Bartholomew's. The next meeting of the committee was held on February 23rd. Sir W. Hart Dyke, M.P., presided, and most of the members of the committee were present. The committee, having heard the evidence of Mr. J. G. Wainwright, treasurer of St. Thomas's Hospital, and two medical practitioners in the City, Dr. F. W. Goodsall and Dr. Great-Rex, adjourned. It resumed its sitting on Friday, February 27th, Sir W. Hart Dyke presiding, and there was a full attendance. The whole of the sitting was occupied in hearing the views of Sir Henry Burdett on the question whether or not it is desirable in the public interest to retain the hospital on its present site. The committee then adjourned until Monday the 2nd inst., when they reassembled at the Mansion House. At this sitting the Lord Mayor presided, and the committee, after a careful consideration of the evidence taken at the four previous sittings, and after a prolonged discussion, passed the following resolution by 14 votes to one :—

> That in the opinion of this committee it is desirable, in the public interest, to retain St. Bartholomew's Hospital on its present site.

It was then decided, on the proposition of the Lord Mayor, to form two sub-committees, the first to deal with buildings, and the second with administration and finance, in order to thoroughly investigate the best means of providing a hospital perfect in every detail and brought up to the latest requirements of modern scientific knowledge on the present site of the hospital in Smithfield. These sub-committees will meet—the former on Mondays and the latter on Fridays—until their investigations are complete.

What the Decision may Mean.

Some confusion has arisen in the public mind in view of the decision arrived at by a majority of 14 to 1 in regard to the site of St. Bartholomew's Hospital. That decision is incomplete as it stands. To understand it, it is essential that a statement should be made in regard to the acreage of the present site, and of the price per square foot per annum at which the governors of St. Bartholomew's Hospital value it. St. Bartholomew's Hospital cannot be made efficient or rebuilt anywhere on a site of less than eight acres. If the governors value the existing site at so little as 1s. to 2s. per square foot per annum, the decision is understandable; if that value is placed at 3s. per square foot or more per annum, much more information is essential to an understanding of the grounds upon which the decision has been come to. Has it been decided to buy further land from Christ's Hospital? The Lord Mayor is reported to have promised to publish the evidence shortly, and when it is forthcoming the foregoing are the points to which attention should be directed in order to clearly understand the issues with a view to forming an accurate judgment on the facts.

